# Electronic Orbital Alignment and Hierarchical Phonon Scattering Enabling High Thermoelectric Performance p-Type Mg_3_Sb_2_ Zintl Compounds

**DOI:** 10.34133/2022/9842949

**Published:** 2022-04-29

**Authors:** Jinsuo Hu, Jianbo Zhu, Fengkai Guo, Haixu Qin, Yijie Liu, Qian Zhang, Zihang Liu, Wei Cai, Jiehe Sui

**Affiliations:** ^1^State Key Laboratory of Advanced Welding and Joining, Harbin Institute of Technology, Harbin 150001, China; ^2^Department of Materials Science and Engineering, Harbin Institute of Technology, Shenzhen 518055, China

## Abstract

Environmentally friendly Mg_3_Sb_2_-based materials have drawn intensive attention owing to their promising thermoelectric performance. In this work, the electrical properties of p-type Mg_3_Sb_2_ are dramatically optimized by the regulation of Mg deficiency. Then, we, for the first time, found that Zn substitution at the Mg2 site leads to the alignment of *p*_*x*,*y*_ and *p*_*z*_ orbital, resulting in a high band degeneracy and the dramatically enhanced Seebeck coefficient, demonstrated by the DFT calculations and electronic properties measurement. Moreover, Zn alloying decreases Mg1 (Zn) vacancies formation energy and in turn increases Mg (Zn) vacancies and optimizes the carrier concentration. Simultaneously, the Mg/Zn substitutions, Mg vacancies, and porosity structure suppress the phonon transport in a broader frequency range, leading to a low lattice thermal conductivity of ~0.47 W m^−1^ K^−1^ at 773 K. Finally, a high *ZT* of ~0.87 at 773 K was obtained for Mg_1.95_Na_0.01_Zn_1_Sb_2_, exceeding most of the previously reported p-type Mg_3_Sb_2_ compounds. Our results further demonstrate the promising prospects of p-type Mg_3_Sb_2_-based material in the field of mid-temperature heat recovery.

## 1. Introduction

Thermoelectric (TE) devices can directly convert heat into electricity and vice versa, which can be used in waste heat recovery and refrigeration applications [1–3]. The conversion efficiency of TE devices (*η*) is determined by the figure of merit of materials (*ZT* = *S*^2^*σT*/*κ*_*tot*_), where *S*, *σ*, *T*, and *κ*_*tot*_ are the Seebeck coefficient, electrical conductivity, absolute temperature, and total thermal conductivity, respectively. The interrelated electrical and thermal transport properties make it difficult to maintain a high power factor (*PF* = *S*^2^*σ*) and a low thermal conductivity simultaneously [4–6]. Previously, several strategies have been proposed to optimize the power factor via band convergence [7, 8], band flatting [9, 10], and energy resonance [11, 12] or reduce the lattice thermal conductivity via grain refinement [13], dense dislocations [14, 15], and hierarchical microstructure [16–18].

Recently, Zintl phase compounds have been extensively investigated as potential mid-temperature TE materials [19–21]. Typical Zintl phases consist of electropositive cations and electronegative polyanions [22, 23]. However, the majority of them contain rare earth elements and or toxic elements, restricting the practical application [24–28]. Recently, Mg_3_Sb_2_-based Zintl materials have drawn wide attention because of the unique attributes of environmentally friendly, low-cost, and abundantly available constituent elements [2, 29–33]. Especially, n-type Mg_3_Sb_2_-based materials have demonstrated excellent TE performance due to the multivalley conduction band behavior near the Fermi level, pushing the necessity of further optimizing the performance of the p-type counterparts that currently have an inferior *ZT* [34–36]. Various dopants (Na, Ag, Li, Zn, Cd, and Mn at the Mg site [37–45], as well as Bi and Pb at the Sb site [46, 47]) have been reported to increase the carrier concentration and thereby improve the TE property of p-type Mg_3_Sb_2_ materials. And Na is very effective for increasing the carrier concentration, but this also leads to increased lattice thermal conductivity [37, 38]. Despite these improvements, the previously reported p-type Mg_3_Sb_2_-based materials still display relatively low *ZT* values, which heavily impede its application potential. Therefore, it is crucial to further promote the TE performance of p-type Mg_3_Sb_2_-based materials given the prerequisite that both n- and p-type legs are required for TE devices. Previous calculations revealed that the valence band edge of Mg_3_Sb_2_ at the Brillouin zone center (Γ) is dominated by the *p* orbitals of Sb anions that are composed of *p*_*x*,*y*_ and *p*_*z*_ orbital [48, 49]. The energy offset of these orbitals is determined as the crystal field splitting energy Δ*E* = *E* (*p*_*x*,*y*_) − *E* (*p*_*z*_). Intuitively, the alignment of these orbitals in the reciprocal space should be straightforward and effective to maximize the band degeneracy (*N*_*v*_) and improve the electronic performance [24, 25, 50]. The TE performance enhancement has been obtained by orbital alignment in YbZn_2_Sb_2_-YbCd_2_Sb_2_, EuZn_2_Sb_2_-EuCd_2_Sb_2_, and MgZn_2_Sb_2_-YbZn_2_Sb_2_-YbMg_2_Sb_2_ Zintl compounds [24, 25, 50].

**Figure 1:**
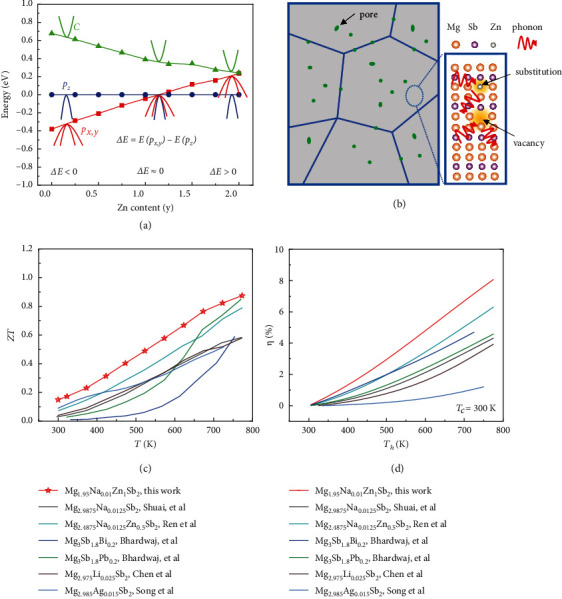
(a) The calculated energy offset of *p*_*x*,*y*_ and *p*_*z*_ orbitals as a function of Zn content *y* in Mg_3-y_Zn_y_Sb_2_. (b) Schematic view of hierarchical microstructure in Mg_2.95-y_Na_0.01_Zn_y_Sb_2_. (c, d) Comparison of *ZT* values and theoretical conversion efficiency of Mg_1.95_Na_0.01_Zn_1_Sb_2_ with some representative p-type Mg_3_Sb_2_ materials [37, 39, 40, 42, 46, 47], respectively.

In this work, Mg_2.99_Na_0.01_Sb_2_ as the base material, Mg vacancies were introduced on the basis of Na acceptor doping to maintain a high carrier concentration and decrease the lattice thermal conductivity simultaneously. More importantly, the isoelectronic substitution of Zn elements was used to manipulate the band structure (Figure 1(a)), leading to the dramatically enhanced density of states (DOS) effective mass and Seebeck coefficient. Meanwhile, the porous structure was formed during the high-temperature sintering process because part of Zn volatilizes as a consequence of its high saturated vapor pressure. The Mg/Zn substitutions, Mg vacancies, and porous structure are responsible for impeding the phonon transport and significantly decreasing the lattice thermal conductivity (Figure 1(b)). Benefited from the increased power factor and significantly decreased thermal conductivity, a high *ZT* value of ~0.87 at 773 K was obtained in Mg_1.95_Na_0.01_Zn_1_Sb_2_, exceeding most of the p-type Mg_3_Sb_2_ materials (Figure 1(c)) [37, 39, 40, 42, 46, 47]. Theoretically, a high predicted efficiency of ~8% was also obtained under the condition of the cold side temperature (*T*_*c*_) at 300 K and the hot side temperature (*T*_*h*_) at 773 K, indicating the promising prospect of our synthesized p-type Mg_3_Sb_2_-based materials (Figure 1(d)). Furthermore, the utilization of tuning the crystal field splitting energy and constructing hierarchical microstructure could also be applied to optimize the TE performance of other Zintl systems.

## 2. Results and Discussion

X-ray diffraction patterns and the calculated lattice parameters of spark plasma sintering (SPS) sintered Mg_2.99-x_Na_0.01_Sb_2_ samples are shown in Figure S1. All the diffraction peaks are indexed to *α*-Mg_3_Sb_2_ with the space group of *P*-3*m*1 without observable impurity peaks. Besides, the change of lattice parameters calculated by the Rietveld refinement is negligible. To further confirm the phase composition, scanning electron microscope (SEM) observations and corresponding energy dispersive spectroscopy (EDS) measurements were performed. As shown in Figure S2, the backscattered electron (BSE) SEM images of the Mg_2.99-x_Na_0.01_Sb_2_ samples confirm that the samples are dense without obvious cracks, consistent with the sample's density measurement (Table S1). The actual compositions determined by EDS are listed in Table S1, and the actual compositions are slightly lower than the nominal ones. The uniform contrast in the BSE micrographs illustrates the single-phase feature when the content of Mg deficiency is less than 0.06, in agreement with the above XRD results. However, some obvious Sb-rich phases begin to appear in the sample of Mg deficiency reaching 0.06.

**Figure 2:**
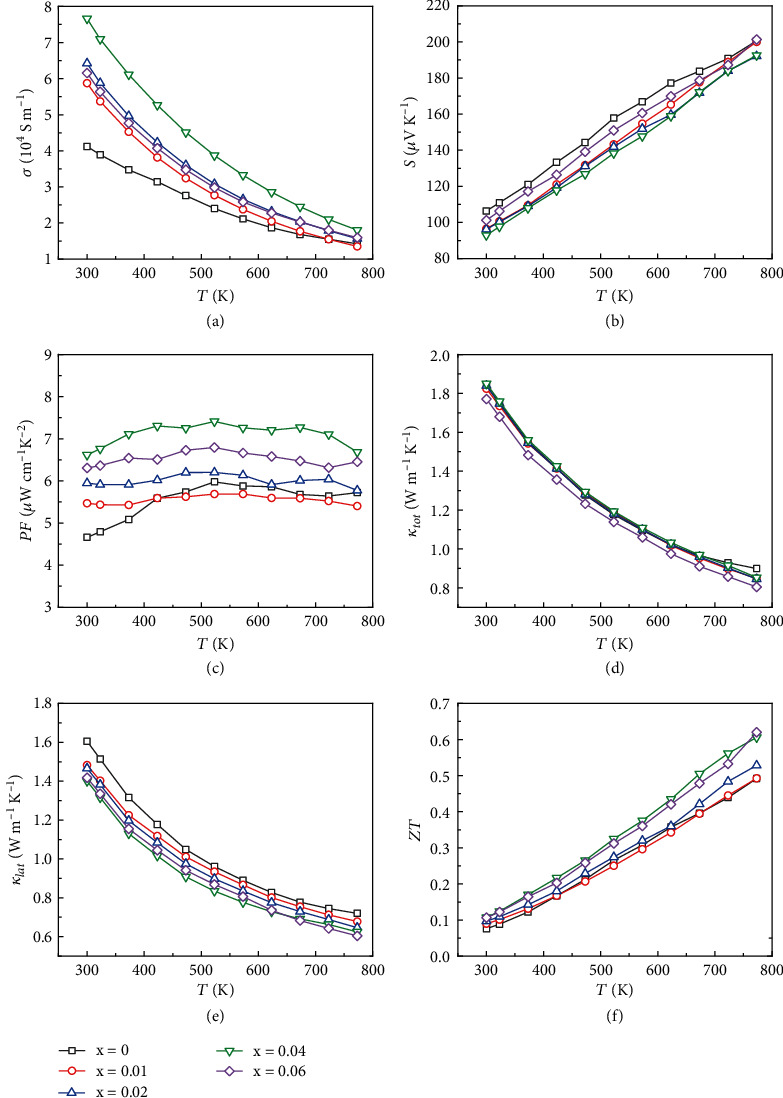
Temperature-dependent thermoelectric transport behavior of Mg_2.99-x_Na_0.01_Sb_2_: (a) electrical conductivity *σ*, (b) Seebeck coefficient *S*, (c) power factor *PF*, (d) total thermal conductivity *κ*_*tot*_, (e) lattice thermal conductivity *κ*_*lat*_, and (f) figure of merit *ZT*.

The temperature-dependent TE properties of Mg_2.99-x_Na_0.01_Sb_2_ are shown in Figure 2. As the Mg deficiency is less than 0.06, the electrical conductivity *σ* increases with increasing the Mg deficiency due to the increased carrier concentration *n*_*H*_, e.g., from 3.80 × 10^19^ cm^−3^ for Mg_2.99_Na_0.01_Sb_2_ sample to 7.26 × 10^19^ cm^−3^ for Mg_2.95_Na_0.01_Sb_2_ sample (Table S1). Simultaneously, the Seebeck coefficient *S* decreases with increasing Mg deficiency. However, when further reducing the Mg content, the anomalous change of both *σ* and *S* is due to the decreased carrier concentration *n*_*H*_ and carrier mobility *μ*_*H*_, which may be related to the obvious precipitation of the Sb-rich phases in this sample. As a consequence of the optimization of *n*_*H*_, the small fraction of Mg deficiency optimizes the electrical properties and enhances the power factor *PF*. Specifically, the *PF* of Mg_2.95_Na_0.01_Sb_2_ sample reaches ~7.3 *μ*W cm^−1^ K^−2^ at 423 K, which is 24% higher than that of the parent material.

The total thermal conductivity *κ*_*tot*_ of samples shows a negligible change with the Mg deficiency content. To understand the phonon-scattering mechanism, the *κ*_*lat*_ is obtained by subtracting the electronic part from the *κ*_*tot*_. The *κ*_*ele*_ can be calculated according to Wiedemann-Franz relation given by *κ_ele_* = *LσT*, where *L* is the Lorenz number estimated by the single parabolic band (SPB) model [51, 52]. As shown in Figure 2(e), the relationship between *κ*_*lat*_ and *T* is almost *T*^−1^, revealing the predominance Umklapp phonon scattering. In general, the *κ*_*lat*_ is reduced with increasing the Mg deficiency owing to the strengthened point defect scattering. The *κ*_*lat*_ of Mg_2.95_Na_0.01_Sb_2_ is 0.62 W m^−1^ K^−1^, which is about 14% lower than that of the parent material. Finally, we obtained the maximum *ZT*~0.64 at 773 K for Mg_2.95_Na_0.01_Sb_2_, a 28% enhancement compared to that of Mg_2.99_Na_0.01_Sb_2_. In principle, the enhanced *ZT* is attributed to the strengthening phonon scattering and the optimized *n*_*H*_ due to the Mg deficiency.

Previous work reported that the Mg2_p_-Sb_p_ bonding dominates the valence band maximum (VBM) of Mg_3_Sb_2_ [49], and the MgSb_4_-tetrahedron unit constitute the anionic [Mg_2_Sb_2_]^2-^ layer. For the undistorted MgSb_4_ tetrahedron, the equivalence of the *x*, *y*,  and *z* directions in the Brillouin zone would lead to triply degenerate *p* orbitals. However, since the distorted MgSb_4_ tetrahedron in the layered Mg_3_Sb_2_ structure, the *p*_*z*_ orbital is separated from the *p*_*x*,*y*_ orbital. To realize high band degeneracy (*N*_*v*_) for enhancing electronic performance, it is relevant for the utilization of orbital engineering (diminish the crystal field splitting energy of orbitals *ΔE*) or achieving very high *n*_*H*_ [48, 50].

**Figure 3:**
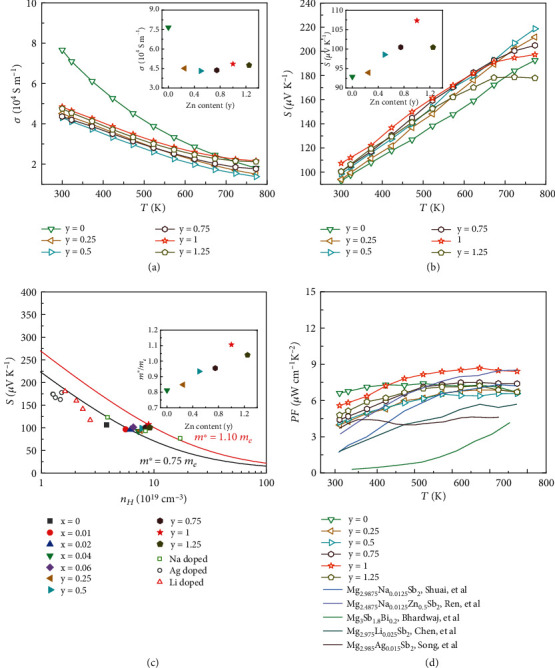
Temperature-dependent electrical properties of Mg_2.95-y_Na_0.01_Zn_y_Sb_2_: (a) electrical conductivity *σ* (the inset shows the room temperature electrical conductivity), (b) Seebeck coefficient *S* (the inset shows the room temperature Seebeck coefficient), (c) carrier concentration *n*_*H*_-dependent Seebeck coefficient *S* of Mg_2.99-x_Na_0.01_Sb_2_, Mg_2.95-y_Na_0.01_Zn_y_Sb_2_ and the previously reported data at room temperature [37, 39, 40], where the solid line was calculated based on the SPB model (the inset shows the DOS effective mass of Mg_2.95-y_Na_0.01_Zn_y_Sb_2_ calculated by the SPB model), and (d) temperature-dependent power factor *PF* of Mg_2.95-y_Na_0.01_Zn_y_Sb_2_ and some representative p-type Mg_3_Sb_2_ materials [37, 39, 40, 42, 46].

Alloying two compounds with opposite Δ*E* signs could manipulate the crystal field splitting energy in 1-2-2 Zintl compounds [24, 48]. Herein, MgZn_2_Sb_2_ with opposite *ΔE* was chosen to alloy with Mg_3_Sb_2_ to tune its band structure [50], and the orbital alignment would occur in MgMg_2-y_Zn_y_Sb_2_ solid solutions. The XRD patterns of Mg_2.95-y_Na_0.01_Zn_y_Sb_2_ samples without obvious peaks of impurities are shown in Figure S3. The EDS mapping of Mg_1.7_Na_0.01_Zn_1.25_Sb_2_ demonstrates the element homogeneity of the samples (Figure S4). The temperature-dependent electrical transport properties of Mg_2.95-y_Na_0.01_Zn_y_Sb_2_ samples are shown in Figure 3. Upon Zn alloying, the room temperature *n*_*H*_ of Mg_2.95-y_Na_0.01_Zn_y_Sb_2_ gradually increases from ~7.26 × 10^19^ cm^−3^ (*y* = 0) to ~9.18 × 10^19^ cm^−3^ (*y* = 1.25) while the *μ*_*H*_ decreases from ~62.80 cm^2^ V^−1^ s^−1^ (*y* = 0) to ~32.34 cm^2^ V^−1^ s^−1^ (*y* = 1.25) (Table S1). The *σ* of all samples decreases with increasing temperature, and this reduction tendency becomes slow at the high-temperature range because of the thermally activated carriers. With increasing the alloying content of Zn, the *σ* first decreases due to the reduced *μ*_*H*_ and then slightly increases due to the enhanced *n*_*H*_ (Table S1). Room temperature *σ* is given in the inset of Figure 3(a) to see the difference more clearly. Considering that the *S* is inversely proportional to the *n*_*H*_, the *S* should decrease with the increased *n*_*H*_. However, the measured room temperature *S* is noticeably improved from 92 to 107 *μ*V K^−1^ after Zn alloying (as shown in the inset of Figure 3(b)). In addition, this critical temperature corresponding to the occurring of the maximum *S* gradually shifts to the lower temperature with increasing the Zn content due to the decreased bandgap.

The SPB model, assuming acoustic phonon scattering as the main mechanism, is commonly used to describe the relationship between the *S* and *n*_*H*_ in TE field [39–41, 53–55]. The experimental data of Mg_2.99-x_Na_0.01_Sb_2_ fitted well with the theoretical prediction line of *m*^∗^ = 0.75 *m*_*e*_ based on the SPB model and was consistent with the previous work [37, 39, 40], indicating the validity of the rigid band approximation when introducing a slight Mg deficiency. However, the experimental data of Mg_2.95-y_Na_0.01_Zn_y_Sb_2_ are above this line, indicating that Zn alloying may change the DOS effective mass and valence band structure of Mg_3_Sb_2_. The DOS effective masses of Mg_2.95-y_Na_0.01_Zn_y_Sb_2_ at room temperature calculated using the SPB model based on the experimental results are shown in the inset of Figure 3(c). The DOS effective mass gradually increases with increasing Zn doping (*y* ≤ 1) which is the underlying reason for the experimentally increased *S*. This is due to the significant change of valence band structure after Zn alloying, leading to the increased *N*_*v*_, which will be thoroughly elaborated in the following discussion. As shown in Figure 3(d), the highest *PF* increases from ~6.7 *μ*W cm^−1^ K^−2^ for Mg_2.95_Na_0.01_Sb_2_ to 8.4 *μ*W cm^−1^ K^−2^ for Mg_1.95_Na_0.01_Zn_1_Sb_2_ due to this beneficial band engineering.

**Figure 4:**
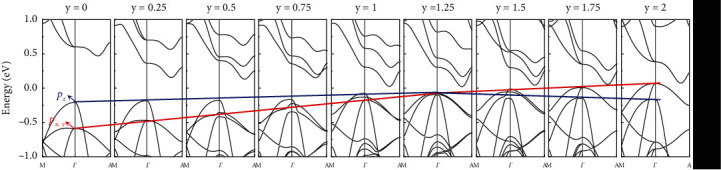
Calculated band structure of Mg_3-y_Zn_y_Sb_2_: *p*_*z*_ orbital gradually converges with *p*_*x*,*y*_ orbitals until the two orbitals diverge at around *y* = 1 with the increasing Zn content (the calculated band structures are built in a 2 × 2 × 1 supercell).

To verify the above analysis, we calculated the band structure of Mg_3-y_Zn_y_Sb_2_, as shown in Figure 4. According to the previous work, Zn atoms are only located in the [Mg_2_Sb_2_]^2-^ layer [56, 57]. With the increasing Zn alloying content, a heavier band contributed from *p_x,y_* orbital of Sb moves much closer to the VBM, so the energy difference Δ*E* of *p*_*x*,*y*_ and *p*_*z*_ orbitals gradually decrease until the two orbitals diverge at around *y* = 1, i.e., alignment of *p*_*x*,*y*_ and *p*_*z*_ orbitals. The orbital alignment is expected to effectively increase *N*_*v*_, resulting in an increased DOS effective mass and *S*. The negligible change *μ*_*H*_ after Zn alloying can be understood by compromise among the introduced defects, involvement of the heavy *p*_*x*,*y*_ orbital, and polarity of the anionic framework [44, 58].

**Figure 5:**
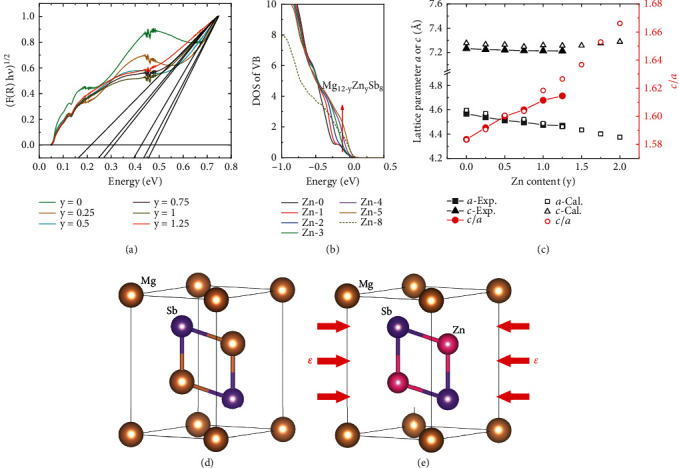
(a) Room-temperature optical absorption versus photo energy for Mg_2.95-y_Na_0.01_Zn_y_Sb_2_. (b) The calculated DOS of Mg_12-y_Zn_y_Sb_8_. (c) The experimental and calculated lattice parameters and the ratio of *c*/*a*. (d, e) The crystal structure of Mg_3_Sb_2_ and MgZn_2_Sb_2_, respectively.

According to the Fourier transport infrared (FTIR) measurement, the estimated optical band gap of Mg_2.95-y_Na_0.01_Zn_y_Sb_2_ at room temperature is 0.48 eV, 0.46 eV, 0.42 eV, 0.31 eV, 0.29 eV, and 0.16 eV for *y* = 0, 0.25, 0.5, 0.75, 1, and 1.25, respectively (Figure 5(a) and Figure S5). The measured optical band gap as a function of Zn content is basically consistent with our previous theoretical calculation value [59, 60]. Figure 5(b) shows that the calculated DOS of Mg_12-y_Zn_y_Sb_8_ increases with the Zn content when the Zn content is less than 5, which is attributed to the gradual alignment of the *p*_*x*,*y*_ and *p*_*z*_ orbitals. Then the sudden drop of DOS of Mg_4_Zn_8_Sb_8_ is due to the orbital diverge (more detailed information is shown in Figure S6).

According to the previous prediction, “the reduced effect from the crystal field on the Mg2-p orbital along the *z*-direction would decrease the energy offset of *p_z_* and *p_x,y_* orbital, and thus lead to the orbital alignment of *p_z_* and *p_x,y_*. Therefore, an intuitive way to achieve the orbital alignment is to enlarge the lattice parameter ratio *c*/*a*. If the atomic distance of *z*-direction is enlarged elongated relative to *x* or *y*, the *p_z_* orbital would suffer a weaker crystal field effect” [49]. As shown in Figure 5(c), lattice parameters *a* and *c* decrease upon Zn doping due to the smaller atomic radius of Zn than Mg, while *a* change more obviously than *c* which is probably related to the inherently layered structure of Mg_3_Sb_2_ [42, 47]. This means that the compressive force is applied in the *ab* plane in the Mg_3_Sb_2_ lattice after Zn doping (Figures 5(d) and 5(e)). According to the previous reports [48], a linear correction between crystal field splitting energy Δ*E* and compressive strain *ε* is observed in the Mg_3_Sb_2_ Zintl compounds. Therefore, applying the compressive biaxial strain in Mg_3_Sb_2_ is more effective for achieving the zero-Δ*E* value, which indicates that the biaxial strain could be used to manipulate the Δ*E* continuously. The maximum *PF* is obtained at the optimal strain corresponding to the minimum Δ*E* according to the first-principles calculations. Therefore, biaxial strain engineering is a valid way to optimize TE performance.

**Figure 6:**
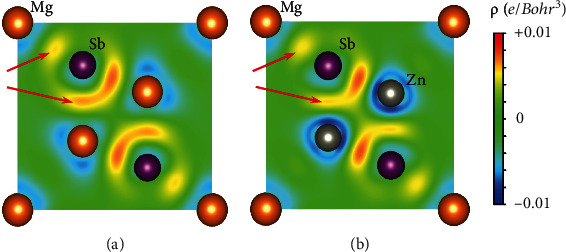
The calculated deformation charge density distribution of (a) Mg_3_Sb_2_ and (b) MgZn_2_Sb_2_.

According to the previous work, the optimal *n*_*H*_ of Mg_3_Sb_2_ gradually increases as the applied compressive stress increases [48]. Zn alloying not only tunes the band structure but also optimizes the *n*_*H*_. As shown in Figure 6, we calculated the deformation charge density distribution of Mg_3_Sb_2_ and MgZn_2_Sb_2_. It is obvious that a small number of electrons gathered between Mg1 and Sb, so it has the characteristic of a weak covalent bond, consistent with the previous work [20, 61]. After a part of Zn replaces at the position of Mg2, the charge accumulation between Mg1 and Sb (Zn and Sb) atoms decreases, which mean that the interaction between atoms becomes weakened. This would cause the Mg1 (Zn) atom to break the local bonding more easily to form vacancies, thereby leading to a higher *n*_*H*_.

**Figure 7:**
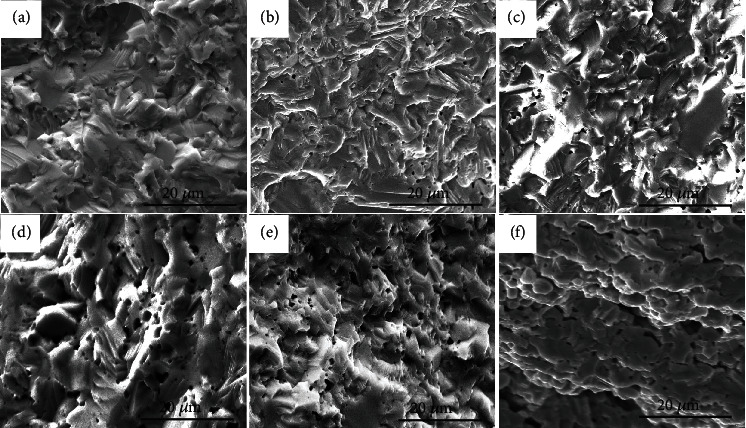
SEM images of Mg_2.95-y_Na_0.01_Zn_y_Sb_2_: (a) *y* = 0, (b) *y* = 0.25, (c) *y* = 0.5, (d) *y* = 0.75, (e) *y* = 1, and (f) *y* = 1.25.

In addition to the beneficial modification on the valence band structure, the fracture surface of the Mg_2.95_Na_0.01_Zn_y_Sb_2_ samples displays the form of brittle transcrystalline fracture with typical lamellar morphology (Figure 7). Moreover, a small number of pores with distinct shapes and different sizes (from nanometers up to a few micrometers) homogeneously distribute in the matrix, while almost no obvious pores are present in the pristine sample, consistent with the sample's density measurement. The diameter of pores size increases gradually with increasing Zn alloying content, while the decreased sample's density is the signature of the formation of pores after Zn alloying. The porous structure may be formed mainly because of part of the Zn volatilization during the high-temperature sintering process, due to its higher saturated vapor pressure and the lower actual content compared to the nominal composition (Table S1). Meanwhile, the volatilization of Mg also plays a part in the formation of the porous structure. The excess amount of element Sb would achieve a eutectic mixture with Mg_3_Sb_2_ [62], so a fraction of liquid phase (Sb and Mg_3_Sb_2_) was squeezed from the bulk sample during the sintering process (Figure S7).

**Figure 8:**
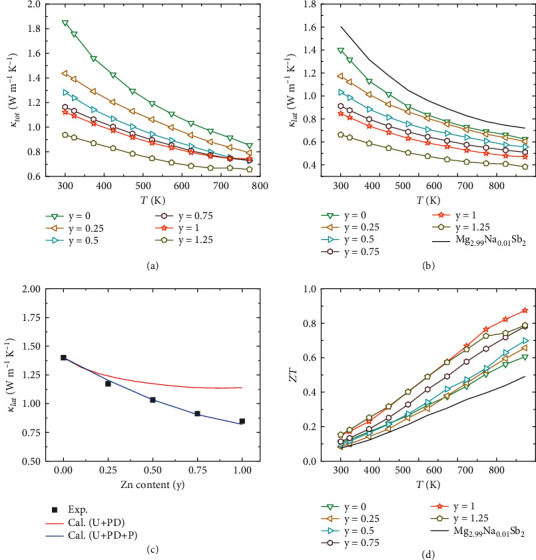
Temperature- or composition-dependent (a) total thermal conductivity *κ*_*tot*_, (b) lattice thermal conductivity *κ*_*lat*_, (c) lattice thermal conductivity *κ*_*lat*_ at room temperature calculated by the Callaway and Klemens model [63, 64, 66, 67], and (d) *ZT* values of Mg_2.95-y_Na_0.01_Zn_y_Sb_2_.

As known, isoelectronic alloying would also lead to a critical reduction of *κ*_*lat*_, due to the strengthened phonon scattering by the introduced mass and strain fluctuations. The temperature-dependent total thermal conductivity *κ*_*tot*_ is shown in Figure 8(a). After Zn alloying, the introduced large mass and negligible strain fluctuations strengthen the point defect scattering. Thus, the *κ*_*tot*_ is significantly reduced due to the suppression of the *κ*_*lat*_ (Figure 8(b)). The *κ*_*lat*_ at 773 K reached 0.38 W m^−1^ K^−1^ in Mg_1.95_Na_0.01_Zn_1_Sb_2_, a 47% reduction compared to Mg_2.99_Na_0.01_Sb_2_.

Under the assumption that the *κ*_*lat*_ is dominant by Umklapp and point defect scattering, the Callaway and Klemens models are widely used in TE field [63–65]. The details of the model and measured sound velocity used to calculate *κ*_*lat*_ are described in the Supporting Information. Based on the *κ*_*lat*_ of the parent compound and the measured sound velocity (*v*), the *κ*_*lat*_ of defected composition with alloying between can be predicted. In correspondence with the decreased *κ*_*lat*_, the scaling parameter (*u*) increases with the increasing content of Zn alloying owing to the increased disorder scaling parameter (Γ_*tot*_). However, the measured *κ*_*lat*_ is lower than the calculated result (Figure 8(c)) which should be attributed to additional phonon scattering from the porous structure. We further analyzed the effectiveness of pores on dampening phonon propagation based on the Callaway model [66, 67]. According to this model, the *κ*_*lat*_ is inversely proportional to the number density of pores *N*_*p*_, which is consistent with our measurement. With increasing Zn alloying content, the relative density decreases; accordingly, the *κ*_*lat*_ for porous samples was gradually reduced due to the increasing number of pores. The Mg vacancies, Mg/Zn substitutions, and porosity structure are responsible for suppressing the phonon transport in a broader frequency range, which significantly diminishes the *κ*_*lat*_. It should be noted that the porous structure strengthened the phonon scattering and reduced the lattice thermal conductivity, but the mechanical property of the samples may also be worsened.

As shown in Figure 8(d), all Zn-alloyed samples show substantially boosted *ZT* due to the alignment of orbitals and the strengthened phonon scattering. In particular, the *ZT* of Mg_1.95_Na_0.01_Zn_1_Sb_2_ sample reaches ~0.87 at 773 K, with an increase of ~78% compared to that of Mg_2.99_Na_0.01_Sb_2_. More importantly, our synthesized sample outperforms most of the previously reported p-type Mg_3_Sb_2_-based materials. In addition, the good reproducibility of three synthesized Mg_1.95_Na_0.01_Zn_1_Sb_2_ samples is confirmed, as shown in the Supporting Information (Figure S8). Repeated tests were also carried out for the Mg_1.95_Na_0.01_Zn_1_Sb_2_ sample, and its TE properties show a negligible change when measuring three times (Figure S9). Indeed, the decreased bonding interaction after Zn alloying may also influence the stability of Mg_3_Sb_2_ material [68–70].

## 3. Conclusions

In conclusion, the current utilization of controlling the Mg deficiency and Zn substitution for Mg significantly enhance the TE performance of p-type Mg_3_Sb_2_. Tuning the Mg deficiency optimizes the carrier concentration and thereby improves the electrical properties. Zn alloying enables an effective orbital alignment for maximizing band degeneracy and optimizing electronic performance. More importantly, the Mg vacancy, Mg/Zn substitution defects, and the porous structure sufficiently suppressed the phonon transport, leading to a remarkable drop of lattice thermal conductivity. Eventually, a dramatically enhanced *ZT* of ~0.87 was obtained for Mg_1.95_Na_0.01_Zn_1_Sb_2_, outperforming most of the previously reported Mg_3_Sb_2_-based materials. Our results demonstrate the effectiveness of using the strategy of orbital alignment and hierarchical structure to independently optimize the thermoelectric property of p-type Mg_3_Sb_2_, which may apply to other thermoelectric systems.

## Data Availability

All data required to support this study are presented in the paper and the supplementary materials. Additional data are available from the authors upon reasonable request.

## Supplementary Materials

Supplementary Materials Experimental section: sample preparation, sample characterization, and electronic structures calculations. Figure S1: (a) X-ray diffraction patterns of Mg_2.99-x_Na_0.01_Sb_2_ samples. (b) Enlarged view between 40° and 50°. (c) Lattice parameter. Figure S2: BSE images of Mg_2.99-x_Na_0.01_Sb_2_: (a) *x* = 0, (b) *x* = 0.01, (c) *x* = 0.02, (d) *x* = 0.04, and (e) *x* = 0.06. Figure S3: X-ray diffraction patterns of Mg_2.95-y_Na_0.01_Zn_y_Sb_2_ samples. Figure S4: SEM image of Mg_1.7_Na_0.01_Zn_1.25_Sb_2_ and corresponding EDS elements mapping. Figure S5: Composition-dependent optical band gap of Mg_2.95-y_Na_0.01_Zn_y_Sb_2_. Figure S6: the calculated DOS of Mg_12-y_Zn_y_Sb_8_ (*y* = 0, 1, 2, 3, 4, 5, 6, 7, and 8). Figure S7: (a) melting-phase XRD pattern of the Na_0.01_Mg_2.2_Zn_0.75_Sb_2_ and Na_0.01_Mg_1.95_Zn_1_Sb_2_ samples and (b, c) the melting phase during the sintering process. Figure S8: repeated measurements on temperature-dependent TE properties of Mg_1.95_Na_0.01_Zn_1_Sb_2_ sample. (a) Electrical conductivity *σ*, (b) Seebeck coefficient *S*, (c) thermal conductivity *κ*_*tot*_, and (d) figure of merit *ZT*. Figure S9: the repeated data of Mg_1.95_Na_0.01_Zn_1_Sb_2_ sample. (a) Electrical conductivity *σ*, (b) Seebeck coefficient *S*, (c) thermal conductivity *κ*_*tot*_, and (d) figure of merit *ZT*. Note S1: phonon modeling studies. Table S1: the measured composition, density, relative density, carrier concentration (*n*_*H*_) and mobility (*μ*_*H*_) of Mg_2.99-x_Na_0.01_Sb_2_ (*x* = 0, 0.01, 0.02, 0.04, and 0.06) and b) Mg_2.95-y_Na_0.01_Zn_y_Sb_2_ (*y* = 0, 0.25, 0.5, 0.75, 1, and 1.25) samples. Table S2: the measured transverse (*v*_*t*_), longitudinal sound velocity (*v*_*l*_), and number density of pores *N*_*p*_ used to calculate *κ*_*lat*_ based on the phonon scattering process for Mg_2.95-y_Na_0.01_Zn_y_Sb_2_ (*y* = 0, 0.25, 0.5, 0.75, 1, and 1.25) samples.
