# Supplementary material for: Electronic Orbital Alignment and Hierarchical Phonon Scattering Enabling High Thermoelectric Performance p-Type Mg3Sb2 Zintl Compounds
Source: Research (Wash D C). 2022 Apr 29;2022:9842949. doi: 10.34133/2022/9842949 (PMC9079362; doi:10.34133/2022/9842949)
Supplement: Supplementary Materials — Experimental section: sample preparation, sample characterization, and electronic structures calculations. Figure S1: (a) X-ray diffraction patterns of Mg2.99-xNa0.01Sb2 samples. (b) Enlarged view between 40° and 50°. (c) Lattice parameter. Figure S2: BSE images of Mg2.99-xNa0.01Sb2: (a) x = 0, (b) x = 0.01, (c) x = 0.02, (d) x = 0.04, and (e) x = 0.06. Figure S3: X-ray diffraction patterns of Mg2.95-yNa0.01ZnySb2 samples. Figure S4: SEM image of Mg1.7Na0.01Zn1.25Sb2 and corresponding EDS elements mapping. Figure S5: Composition-dependent optical band gap of Mg2.95-yNa0.01ZnySb2. Figure S6: the calculated DOS of Mg12-yZnySb8 (y = 0, 1, 2, 3, 4, 5, 6, 7, and 8). Figure S7: (a) melting-phase XRD pattern of the Na0.01Mg2.2Zn0.75Sb2 and Na0.01Mg1.95Zn1Sb2 samples and (b, c) the melting phase during the sintering process. Figure S8: repeated measurements on temperature-dependent TE properties of Mg1.95Na0.01Zn1Sb2 sample. (a) Electrical conductivity σ, (b) Seebeck coefficient S, (c) thermal conductivity κtot, and (d) figure of merit ZT. Figure S9: the repeated data of Mg1.95Na0.01Zn1Sb2 sample. (a) Electrical conductivity σ, (b) Seebeck coefficient S, (c) thermal conductivity κtot, and (d) figure of merit ZT. Note S1: phonon modeling studies. Table S1: the measured composition, density, relative density, carrier concentration (nH) and mobility (μH) of Mg2.99-xNa0.01Sb2 (x = 0, 0.01, 0.02, 0.04, and 0.06) and b) Mg2.95-yNa0.01ZnySb2 (y = 0, 0.25, 0.5, 0.75, 1, and 1.25) samples. Table S2: the measured transverse (vt), longitudinal sound velocity (vl), and number density of pores Np used to calculate κlat based on the phonon scattering process for Mg2.95-yNa0.01ZnySb2 (y = 0, 0.25, 0.5, 0.75, 1, and 1.25) samples. [file 9842949.f1.docx]

**Supporting Information**

# Electronic Orbital Alignment and Hierarchical Phonon Scattering Enabling High Thermoelectric Performance p-Type Mg_3_Sb_2_ Zintl Compounds

Jinsuo Hu, ^1^ Jianbo Zhu, ^1^ Fengkai Guo, ^1^ Haixu Qin, ^1^ Yijie Liu, ^2^ Qian Zhang, ^2^ Zihang Liu, ^1,*^ Wei Cai, ^1^ and Jiehe Sui ^1,*^

^1^State Key Laboratory of Advanced Welding and Joining, Harbin Institute of Technology, Harbin 15000, China

^2^Department of Materials Science and Engineering, Harbin Institute of Technology, Shenzhen 518055, China

Correspondence should be addressed to Zihang Liu zihangliu@hit.edu.cn; and Jiehe Sui; suijiehe@hit.edu.cn

# Experiment Section

*Sample preparation*: Stoichiometric amounts of high purity Magnesium turnings (Mg, 99.95%), sodium cubes (Na, 99.9%), antimony shots (Sb, 99.9%), and zinc powder (Zn, 99.9%) were weighted according to the compositions of Mg_2.99-x_Na_0.01_Sb_2_ (x = 0, 0.01, 0.02, 0.04, and 0.06) and Mg_2.95-y_Na_0.01_Zn_y_Sb_2_ (y = 0.25, 0.5, 0.75, 1, and 1.25) in the glove box with Ar atmosphere, and then loaded into a stainless-steel jar and ball milled for 10 h by a high energy ball mill (SPEX 8000M). The obtained powder was then loaded into a graphite die with an inner diameter of 12.7 mm, and sintered instantly by spark plasma sintering (SPS) at 973 K for 2 min under the pressure of 50 MPa.

*Structure characterizations*: The phase composition of the samples was characterized by X-ray diffraction (PANalytical diffractometer with a Cu Kα1 radiation source) (X-ray diffraction (PANalytical diffractometer with a Cu Kα1 radiation source) was used to examined crystal structure of the samples). The microstructure and composition analyses were investigated by scanning electron microscopy (SEM) of FEI Quanta 200FEG equipped with an energy dispersive spectroscopy (EDS).

*Electronic structure calculations*: The electronic structures were computed using Vienna Ab initio Simulations Package (VASP) with projector-augmented wave pseudopotentials [1]. As for exchange-correlation, Tran-Blaha-modified Becke-Johnson (mBJ) functional was used after pre-convergence with the Perdew-Burke-Ernzerhof (PBE) functional [2, 3]. The structures of random substitutional alloy are built by VASPKIT software in a 2×2×1 supercell (Mg_12-y_Zn_y_Sb_8_, 0≤y≤8) [4]. A plane wave cutoff energy of 550 eV and a 9×9×7 k-mesh were used for atomic coordinates optimizations.

*Thermoelectric transport properties measurements*: Both electrical conductivity (*σ*) and Seebeck coefficient (*S*) were simultaneously measured under the helium atmosphere from room temperature to 773 K on a commercial system (ZEM-3, ULVAC Riko). The total thermal conductivity, *κ_tot_* = *ρ* *D* *C*_p_ was calculated using the measured volumetric density (*ρ*) by the Archimedes method, thermal diffusivity (*D*) by the laser flash apparatus (LFA457, NETZSCH), and heat capacity (*C_p_*) estimated according to the Dulong-Petit relationship *C_p_* = 3 *NR*/*M_w_*, where *N* is the atom number per formula unit, *M_w_* is the molecular weight of the considered formula unit, and *R* is the gas constant. The Hall coefficient (*R_H_*) was conducted using the van der Pauw method under a reversible magnetic field of 1.5 T. The carrier concentration (*n_H_*) and the carrier mobility (*μ_H_*) were calculated by *n_H_* = 1/*e* *R_H_* and *μ_H_* = *σ* *R_H_* respectively, where *e* is the elementary charge. The room temperature bandgap was measured using the Fourier Transform Infrared (FTIR) Spectrometer system (iS50, Thermo Fisher Nicolet).


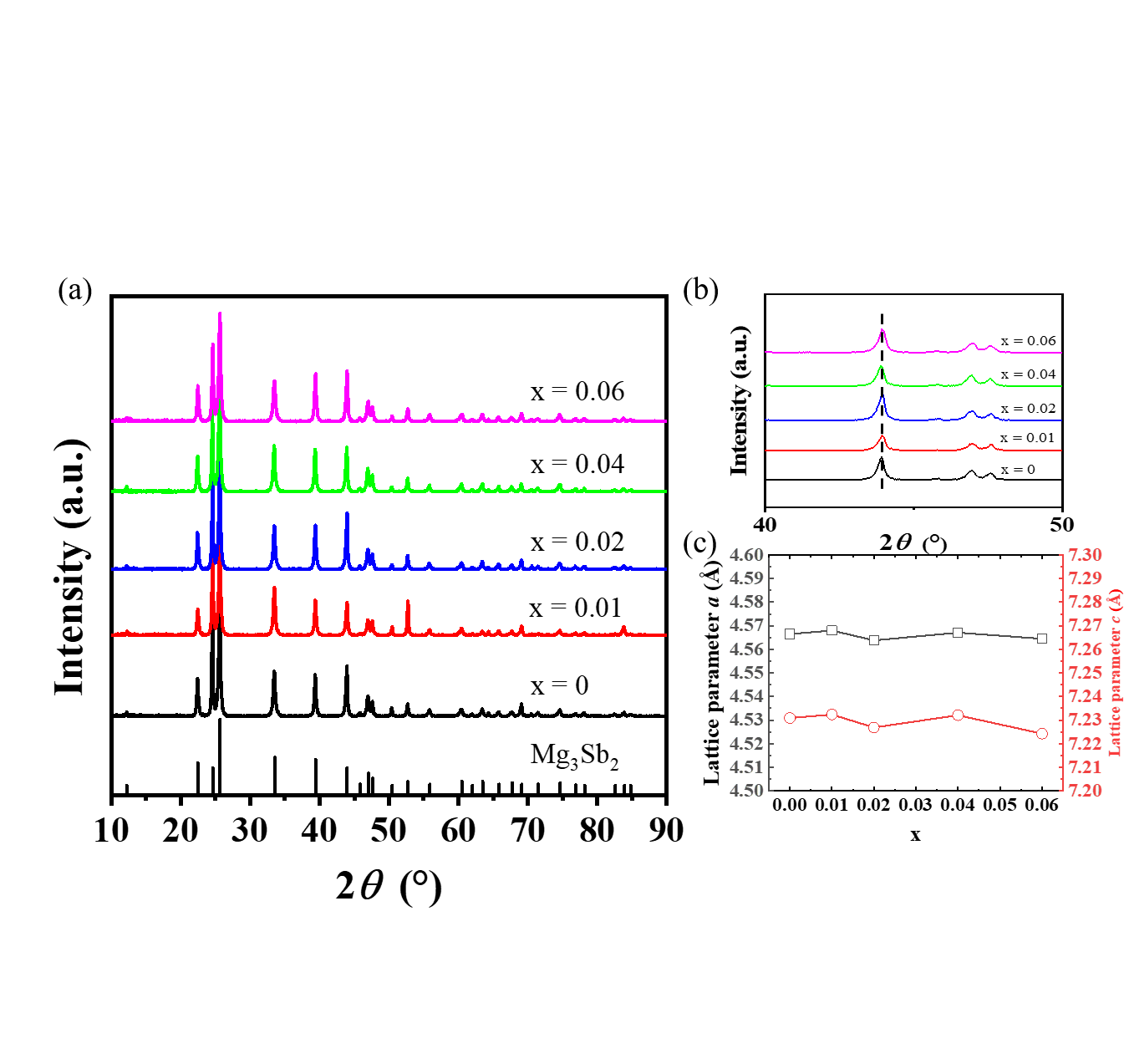


**Figure S1**. (a) X-ray diffraction patterns of Mg_2.99-x_Na_0.01_Sb_2_ samples. (b) Enlarged view between 40° and 50°. (c) Lattice parameter.


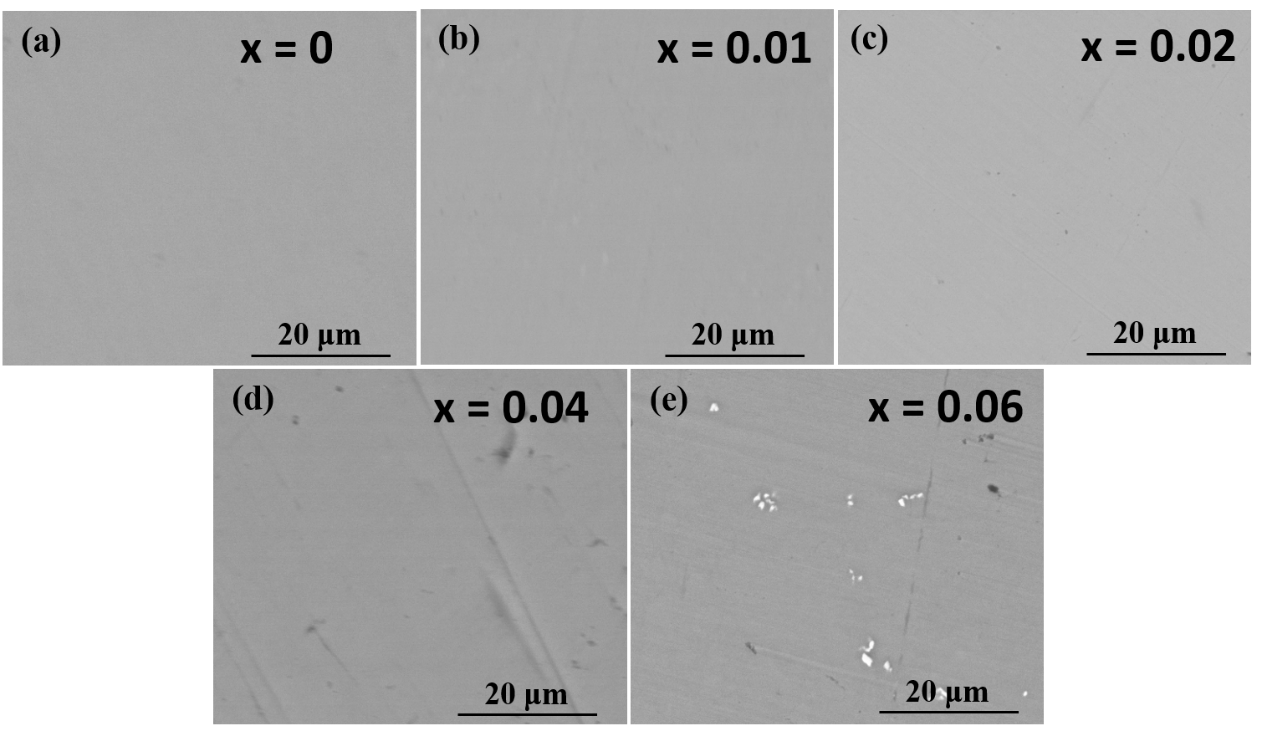


**Figure S2**. BSE images of Mg_2.99-x_Na_0.01_Sb_2_, (a) x = 0, (b) x = 0.01, (c) x = 0.02, (d) x = 0.04, (e) x = 0.06.


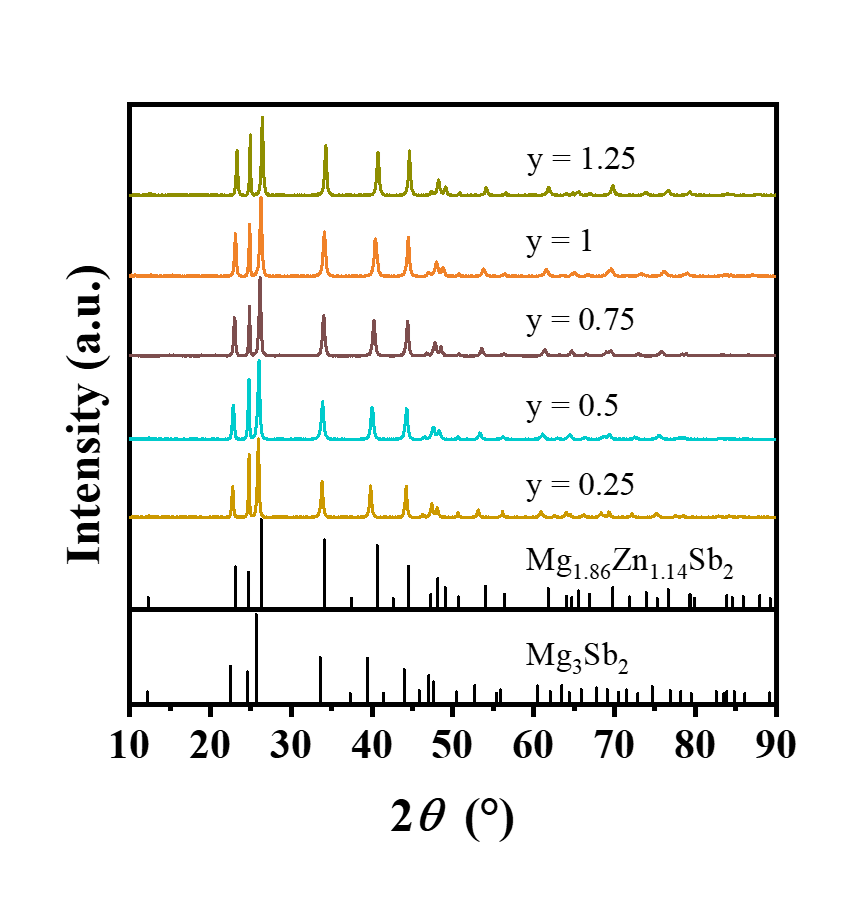


**Figure S3**. X-ray diffraction patterns of Mg_2.95-y_Na_0.01_Zn_y_Sb_2_ samples.


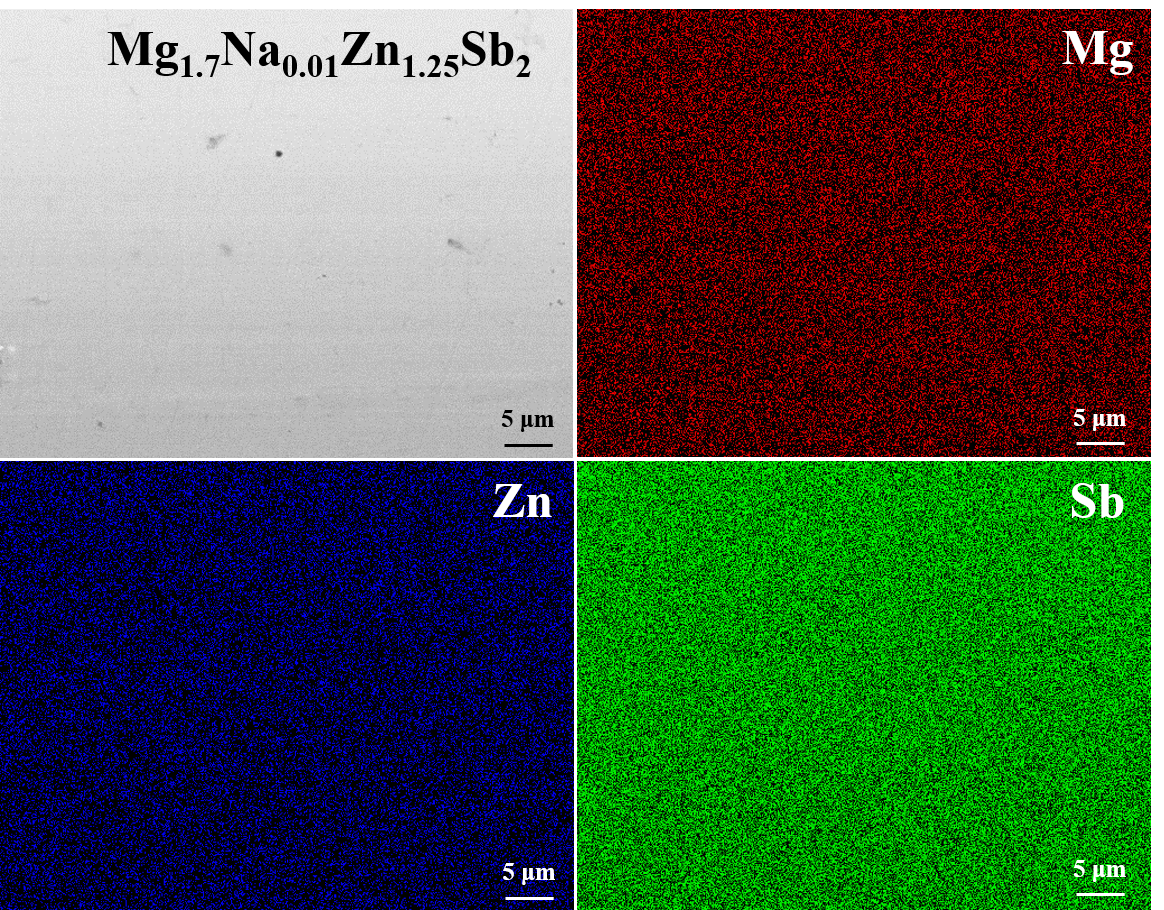


**Figure S4**. SEM image of Mg_1.7_Na_0.01_Zn_1.25_Sb_2_ and corresponding EDS elements mapping.


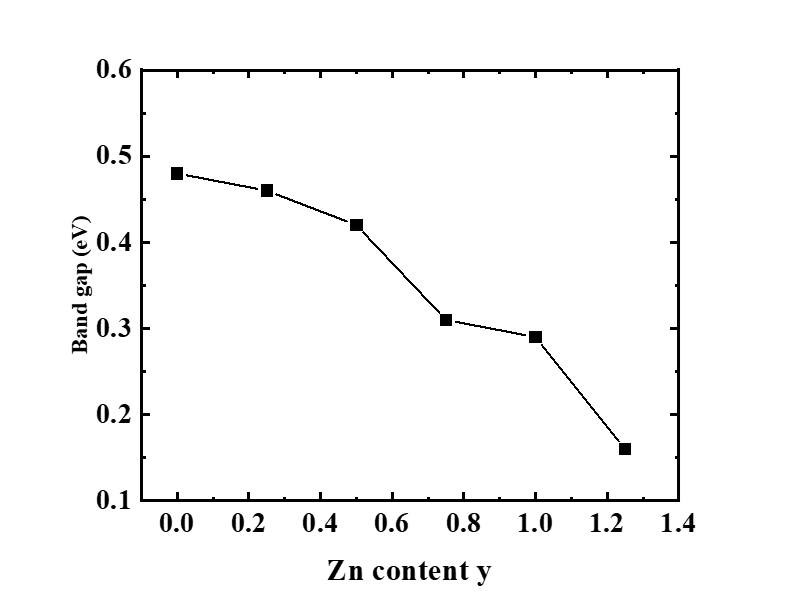


**Figure S5**. Composition-dependent optical band gap of Mg_2.95-y_Na_0.01_Zn_y_Sb_2_.


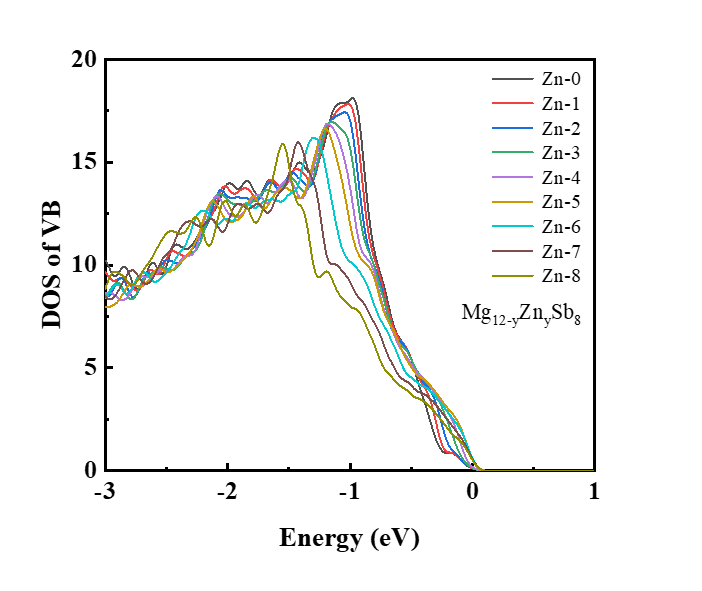


**Figure S6**. The calculated DOS of Mg_12-y_Zn_y_Sb_8_ (y = 0, 1, 2, 3, 4, 5, 6, 7, and 8).


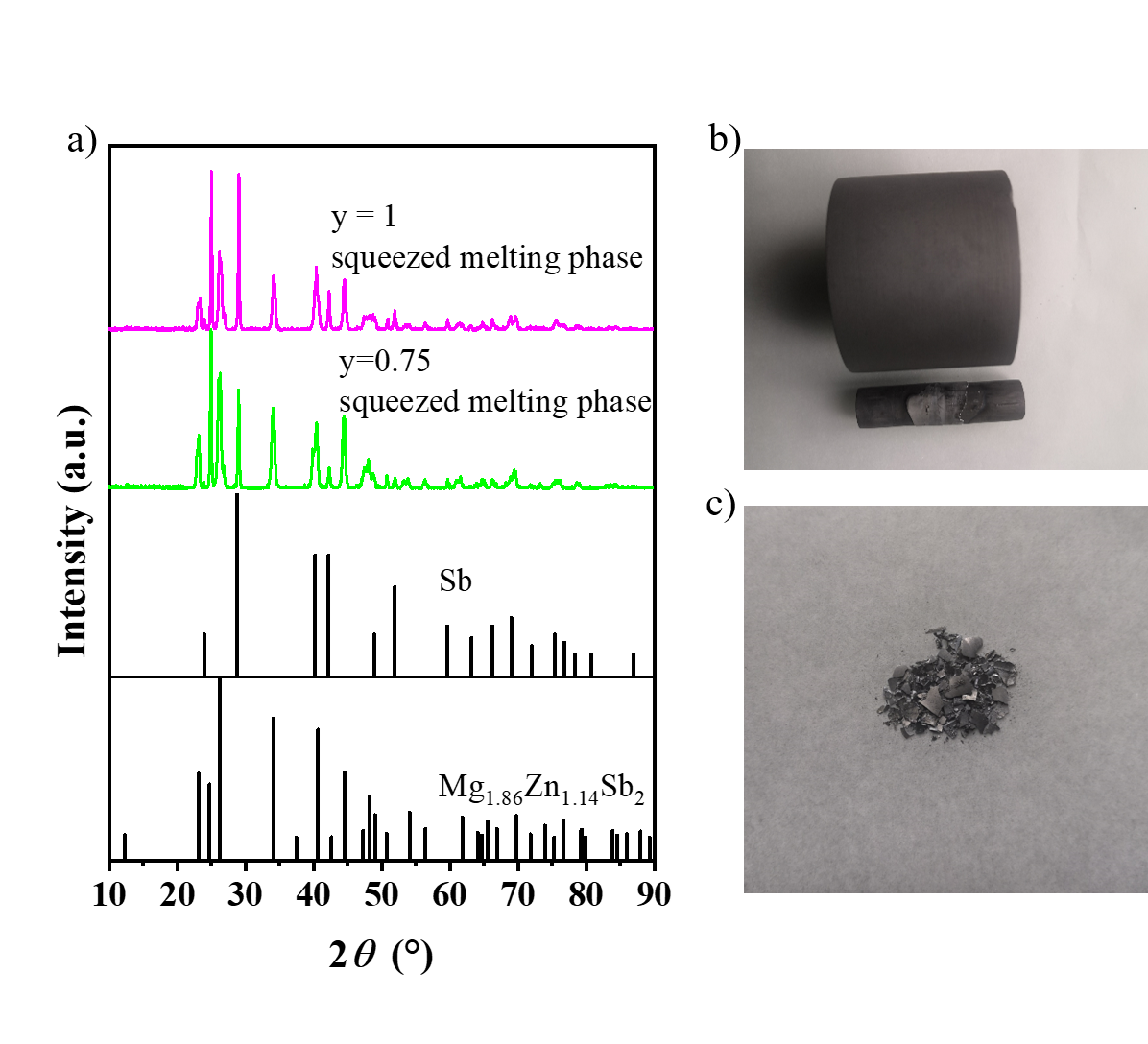


**Figure S7**. (a) melting-phase XRD pattern of the Na_0.01_Mg_2.2_Zn_0.75_Sb_2_ and Na_0.01_Mg_1.95_Zn_1_Sb_2_ samples, (b) and (c) the melting phase during the sintering process.


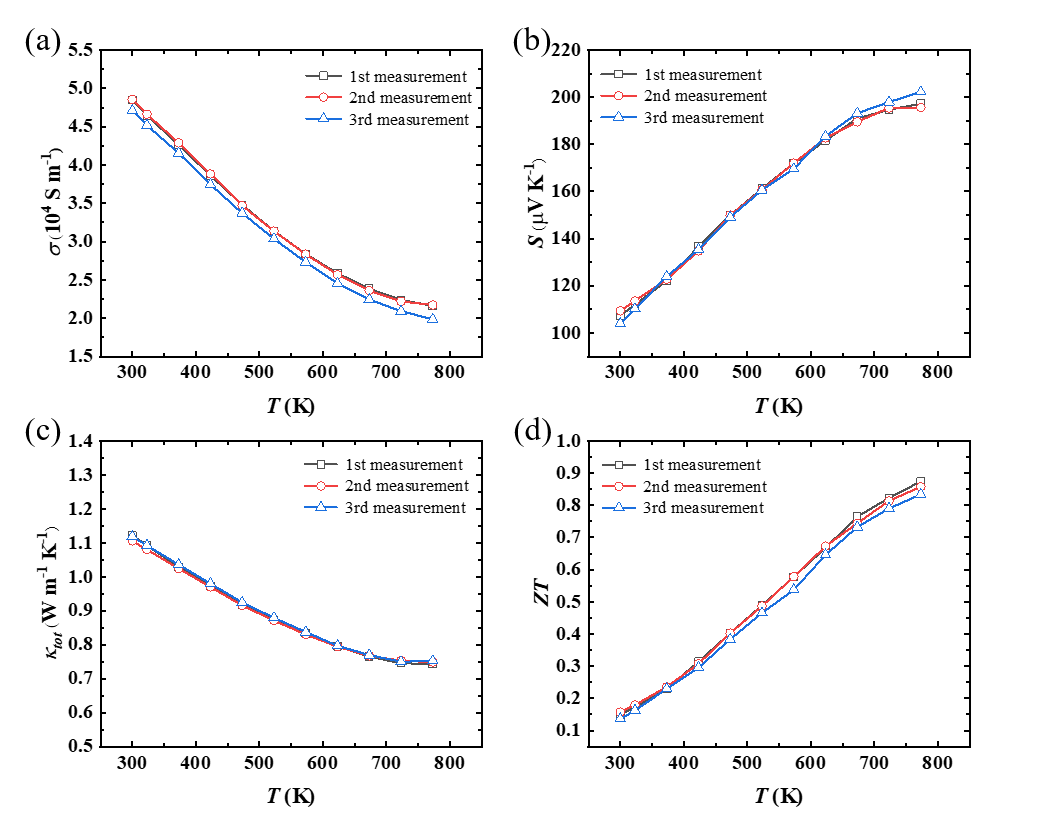


**Figure S8**. Repeated measurements on temperature dependent TE properties of Mg_1.95_Na_0.01_Zn_1_Sb_2_ sample. (a) Electrical conductivity *σ*, (b) Seebeck coefficient *S*, (c) thermal conductivity *κ_tot_*, and (d) figure of merit *ZT*.


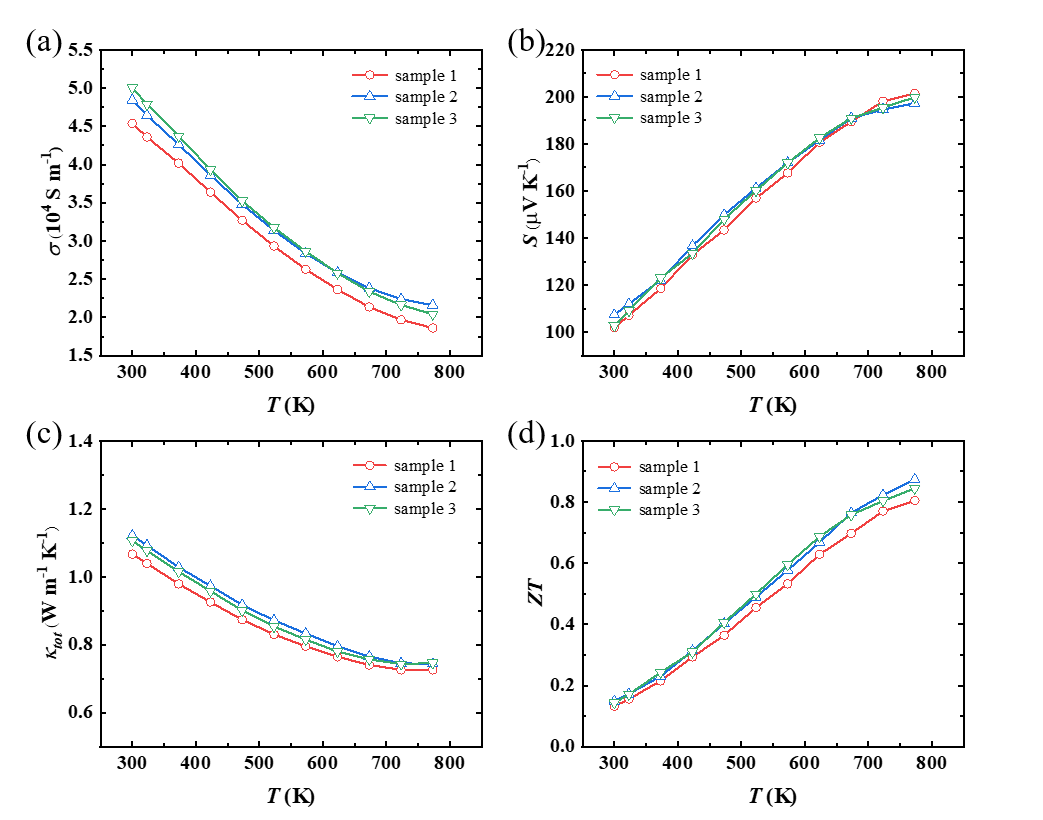


**Figure S9**. The repeated data of Mg_1.95_Na_0.01_Zn_1_Sb_2_ sample. (a) Electrical conductivity *σ*, (b) Seebeck coefficient *S*, (c) thermal conductivity *κ_tot_*, and (d) figure of merit *ZT*.

**Note 1: Phonon modeling studies**

Based on the Callaway and Klemens model [5, 6], we analyzed the correlation between the point defect scattering induced by Zn-doping and *κ_lat_*. Under the assumption that the *κ_lat_* is dominant by Umklapp and point defect scattering, this model proposed a relationship between the lattice thermal conductivity of an alloyed compound (*κ_lat_*) and of a pure compound used in that alloy (*κ_lat, p_*)

$\frac{\kappa_{lat}}{\kappa_{lat, p}}=\frac{{tan}^{-1}(u)}{u}$ (S1)

$u={(\frac{\pi^{2}\theta_{D}\Omega}{hv_{a}^{2}}\kappa_{lat,p}\Gamma_{tot})}^{1/2}$ (S2)

where *u* is a scaling parameter, *Θ_d_* is the Debye temperature, *Ω* is the average volume per atom, *Γ_tot_* is the disorder scaling parameter, *h* is Planck constant, and *v_a_* is the average sound velocity given by

$v_{a}={(\frac{1}{3}(\frac{1}{v_{l}^{3}}+\frac{2}{v_{t}^{3}}))}^{{-1}/3}$ (S3)

here, the longitudinal (*v_l_*) and transverse (*v_t_*) sound velocity were experimentally measured and shown in **Table S2**. The Debye temperature *Θ_d_* was calculated using the sound velocity by

$\theta_{D}={\frac{h}{k_{B}}(\frac{3N}{4\pi V})}^{1/3}v_{a}$ (S4)

where *N* is the number of atoms in a unit cell and *V* is the unit cell volume. The disorder scaling parameter can be calculated by summing its mass fluctuations (*Γ_m_*) and strain fluctuations (*Γ_s_*) contribution

$\Gamma_{m}=\frac{\sum_{i=1}^{n} c_{i}\left( \frac{\bar{M}}{M} \right)^{2}f_{1}f_{2}{(\frac{M_{1}-M_{2}}{\bar{M}})}^{2}}{\sum_{i=1}^{n} c_{i}}$ (S5)

$\Gamma_{s}=\frac{\sum_{i=1}^{n} c_{i}\left( \frac{\bar{M}}{M} \right)^{2}f_{1}f_{2}{\varepsilon(\frac{r_{1}-r_{2}}{\bar{r}})}^{2}}{\sum_{i=1}^{n} c_{i}}$ (S6)

$\bar{M}=f_{1}M_{1}+f_{2}M_{2}$, $\bar{r}=f_{1}r_{1}+f_{2}r_{2}$ (S7)

$M=(\frac{\sum_{i=1}^{n} c_{i}\bar{M}}{\sum_{i=1}^{n} c_{i}})$ (S8)

where *M* is the atomic mass, *r* is the atomic radius, *f* is the fractional occupancies at the Mg2 sublattice, *c_i_* is the total occupancy of the ith sublattice. *ε* is the strain field factor, which can be estimated by the following equations:

$\varepsilon=\frac{2}{9}{(\frac{6.4\times\gamma(1+\upsilon_{p})}{(1-\upsilon_{p})})}^{2}$ (S9)

$\upsilon_{p}=\frac{1-2{({v_{s}}/{v_{l}})}^{2}}{2-2{({v_{s}}/{v_{l}})}^{2}}$ (S10)

$\gamma=\frac{3}{2}(\frac{1+\upsilon_{p}}{2-3\upsilon_{p}})$ (S11)

where *γ* is the Gruneisen parameter, *υ_p_* is the Poisson ratio.

We further analyzed the effectiveness of pores on dampening phonon propagation based on the Callaway model [7, 8], the *κ_lat_* is given by

$\kappa_{lat}=\frac{{4\pi k}_{B}}{v_{a}}{(\frac{k_{B}T}{h})}^{3}\int_{0}^{{\theta_{D}}/T} \tau_{tot}\frac{x^{4}e^{x}}{{(e^{x}-1)}^{2}}dx$ (S12)

where *k*_B_ is the Boltzmann constant, $x=\frac{h\omega}{2\pi k_{B}T}$ is the normalized frequency, *τ_tot_* is the total relaxation time. For the scattering of pores or nano-precipitates, the relaxation time *τ_p_* is determined by

${\tau_{p}}^{-1}=v({\sigma_{s}}^{-1}+{\sigma_{l}}^{-1})N_{P}$ (S13)

$\sigma_{s}=2\pi R^{2}$ (S14)

$\sigma_{l}=\frac{4}{9}{\pi R^{2}(\frac{\Delta D}{D})}^{2}{(\frac{\omega R}{v})}^{4}$ (S15)

where *D* is the radius of the pores or nano-precipitates, *ΔD* is the density difference between pores/nano-precipitates and the host material, and *N_p_* is the number density of pores/nano-precipitates pores (similar to pore volume or relative porosity). In porous materials, the *τ_p_* is calculated by assuming *ΔD* equal to *D*.

**Table S1**. The measured composition (To facilitate comparison, each element composition is converted on the fixed Sb amount of 2), density, relative density, carrier concentration (*n_H_*) and mobility (*μ_H_*) of Mg_2.99-x_Na_0.01_Sb_2_ (x = 0, 0.01, 0.02, 0.04, and 0.06) and b) Mg_2.95-y_Na_0.01_Zn_y_Sb_2_ (y = 0, 0.25, 0.5, 0.75, 1, and 1.25) samples.

| Nominal composition | Actual composition | Density  (g/cm^3^) | Relative density (%) | *n_H_*  (10^19^ cm^-3^) | *μ_H_*  (cm^2^V^-1^s^-1^) |
| --- | --- | --- | --- | --- | --- |
| Mg_2.99_Na_0.01_Sb_2_ | Mg_2.74_Sb_2_ | 3.950 | 98.3 | 3.80 | 67.77 |
| Mg_2.98_Na_0.01_Sb_2_ | Mg_2.75_Sb_2_ | 3.976 | 98.9 | 5.60 | 65.56 |
| Mg_2.97_Na_0.01_Sb_2_ | Mg_2.71_Sb_2_ | 3.978 | 99.0 | 6.20 | 64.78 |
| Mg_2.95_Na_0.01_Sb_2_ | Mg_2.70_Sb_2_ | 3.975 | 98.9 | 7.26 | 62.80 |
| Mg_2.93_Na_0.01_Sb_2_ | Mg_2.74_Sb_2_ | 3.976 | 98.9 | 6.56 | 58.61 |
| Mg_2.7_Na_0.01_Zn_0.25_Sb_2_ | Mg_2.50_Zn_0.22_Sb_2_ | 4.130 | 98.1 | 7.60 | 37.05 |
| Mg_2.45_Na_0.01_Zn_0.5_Sb_2_ | Mg_2.26_Zn_0.46_Sb_2_ | 4.254 | 96.8 | 7.80 | 34.49 |
| Mg_2.2_Na_0.01_Zn_0.75_Sb_2_ | Mg_2.09_Zn_0.66_Sb_2_ | 4.336 | 95.0 | 8.45 | 32.26 |
| Mg_1.95_Na_0.01_Zn_1_Sb_2_ | Mg_2.03_Zn_0.78_Sb_2_ | 4.390 | 92.5 | 8.96 | 33.78 |
| Mg_1.7_Na_0.01_Zn_1.25_Sb_2_ | Mg_1.56_Zn_0.98_Sb_2_ | 4.414 | 90.1 | 9.18 | 32.34 |

**Table S2**. The measured transverse (*v_t_*), longitudinal sound velocity (*v_l_*), and number density of pores *N_p_* used to calculate *κ_lat_* based on the phonon scattering process for Mg_2.95-y_Na_0.01_Zn_y_Sb_2_ (y = 0, 0.25, 0.5, 0.75, 1, and 1.25) samples.

| Sample | y = 0 | y = 0.25 | y = 0.5 | y = 0.75 | y = 1 | y = 1.25 |
| --- | --- | --- | --- | --- | --- | --- |
| *v*_t_ (m s^-1^) | 1759.49 | 1972.79 | 2013.69 | 1934.64 | 1935.89 | 1831.32 |
| *v*_l_ (m s^-1^) | 4276.92 | 4142.85 | 3868.42 | 3474.17 | 3578.19 | 3009.90 |
| *v*_a_ (m s^-1^) | 1991.27 | 2219.04 | 2253.33 | 2154.31 | 2160.47 | 2023.08 |
| *R* (nm) | 0 | 500 | 625 | 750 | 1 | - |
| *N_p_* (10^21^ m^-3^) | 0 | 2.49 | 18.98 | 50.54 | 108.6 | - |

References

[1] G. Kresse and J. Hafner, "Ab initio molecular dynamics for liquid metals," *Physical Review B*, vol. 47, no. 1, pp. 558-561, 1993.

[2] J.P. Perdew, K. Burke, and M. Ernzerhof, "Generalized gradient approximation made simple," *Physical Review Letters*, vol. 77, no. 18, pp. 3865-3868, 1996.

[3] A.D. Becke and E.R. Johnson, "A simple effective potential for exchange," *Journal of Chemical Physics*, vol. 124, 221101, 2006.

[4] V. Wang, N. Xu, J.-C. Liu, G. Tang, W.-T. Geng, "VASPKIT: a user-friendly interface facilitating high-throughput computing and analysis using VASP code," *Computer Physics Communications*, vol. 267, article 108033, 2021.

[5] P.G. Klemens, "Thermal resistance due to point defects at high temperatures," *Physical Review*, vol. 119, no. 2, pp. 507-509, 1960.

[6] M. Wood, U. Aydemir, S. Ohno, and G.J. Snyder, "Observation of valence band crossing: the thermoelectric properties of CaZn_2_Sb_2_–CaMg_2_Sb_2_ solid solution," *Journal of Materials Chemistry A*, vol. 6, no. 20, pp. 9437-9444, 2018.

[7] J. Callaway and H.C. von Baeyer, "Effect of point imperfections on lattice thermal conductivity," *Physical Review*, vol. 120, no. 4, pp. 1149-1154, 1960.

[8] M. Hong, Y. Wang, S. Xu et al., "Nanoscale pores plus precipitates rendering high-performance thermoelectric SnTe_1-x_Se_x_ with refined band structures," *Nano Energy*, vol. 60, pp. 1-7, 2019.
